# Evaluation of the frequency of neuroimaging findings in congenital infection by Zika virus and differences between computed tomography and magnetic resonance imaging in the detection of alterations

**DOI:** 10.1590/0037-8682-0557-2019

**Published:** 2020-11-25

**Authors:** Bruno Niemeyer de Freitas Ribeiro, Bernardo Carvalho Muniz, Edson Marchiori

**Affiliations:** 1 Hospital Casa de Portugal / 3D Diagnóstico por Imagem, Departamento de Radiologia, Rio de Janeiro, RJ, Brasil.; 2 Instituto Estadual do Cérebro Paulo Niemeyer, Departamento de Radiologia, Rio de Janeiro, RJ, Brasil.; 3 Universidade Federal do Rio de Janeiro, Departamento de Radiologia, Rio de Janeiro, RJ, Brasil.

**Keywords:** Zika virus, Neuroimaging, Congenital, Magnetic resonance imaging, X-Ray Computed tomography, Communicable diseases

## Abstract

**INTRODUCTION:**

Congenital infection by the Zika virus (ZIKV) is responsible for severe abnormalities in the development of the central nervous system. The aim of this study was to evaluate and compare the ability of computed tomography (CT) and magnetic resonance (MR) to detect patterns of involvement of the central nervous system in congenital ZIKV syndrome.

**METHODS:**

We retrospectively analyzed CT and MR images from 34 patients with congenital ZIKV syndrome and evaluated the differences between the two methods in detecting alterations.

**RESULTS:**

The predominant radiographic finding was a simplified gyral pattern, present in 97% of cases. The second most common finding was the presence of calcifications (94.1%), followed by ventriculomegaly (85.3%), dysgenesis of the corpus callosum (85.3%), craniofacial disproportion and redundant scalp (79.4%), complete opercular opening (79.4%), occipital prominence (44.1%), cerebellar hypoplasia (14.7%), and pontine hypoplasia (11.8%). The gyral pattern was extensively simplified in most cases, and calcifications were located predominantly at the cortical-subcortical junction. CT was able to better identify calcifications (94.1% × 88.2%), while MRI presented better spatial resolution for the characterization of gyral pattern (97% × 94.1%) and corpus callosum dysgenesis (85.3% × 79.4%).

**CONCLUSIONS:**

Although congenital ZIKV syndrome does not present pathognomonic neuroimaging findings, some aspects, such as calcifications at the cortical-subcortical junction, especially when associated with compatible clinical and laboratory findings, are suggestive of intrauterine ZIKV infection.

## INTRODUCTION

Flaviviruses transmitted by mosquitoes and ticks are among the most important emerging viruses. The Zika virus (ZIKV) pandemic is the most recent emerging viral disease transmitted by arthropods. Outbreaks of dengue, West Nile fever, and chikungunya occurred in 1990, 1999, and 2013, respectively^1^.

Computed tomography (CT) and magnetic resonance (MR) imaging are of great importance in the diagnosis of congenital ZIKV syndrome. Although neuroimaging findings are not pathognomonic, many are very suggestive of ZIKV involvement. The objective of this study was to describe and compare the most frequent radiological findings of congenital ZIKV syndrome on CT and MR imaging of the brain, by analyzing images of 34 patients with this condition.

## METHODS

For this observational, descriptive, and retrospective study, brain CT and MR images from 49 patients with suspected congenital ZIKV syndrome who were referred to the Instituto Estadual do Cérebro Paulo Niemeyer, Rio de Janeiro, Brazil, were examined. All patients received adequate prenatal monitoring and care, including maternal testing for toxoplasmosis, syphilis, and rubella, among other infectious and parasitic diseases. Diabetic and hypertensive mothers were properly monitored and had adequate glycemic and blood pressure control during the prenatal period. There was no history of smoking or drinking during pregnancy.

Images from 15 patients were excluded due to normal findings (*n* = 11), post-surgical status (*n* = 2), and the presence of patterns that were not compatible with congenital ZIKV syndrome (*n* = 2; hypoxic-ischemic insult and perinatal hemorrhage, respectively). The Ethics and Research Committee of the Instituto Estadual do Cérebro Paulo Niemeyer and the University Hospital Clementino Fraga Filho of the Federal University of Rio de Janeiro approved the study. The researcher followed the principles of the Declaration of Helsinki.

The diagnosis of congenital ZIKV syndrome was based on a compatible clinical history, radiological findings reflecting typical disease patterns, and positivity on serological tests (enzyme-linked immunosorbent assay for immunoglobulin M and G antibodies to ZIKV).

CT and MR imaging examinations were performed on the same devices (Siemens Somaris Emotion 16 and Siemens Magnetom Avanto 1.5T, Erlangen, Germany). Axial slices were acquired from the apex to the base of the skull. All MR examinations were performed under sedation. Cranial CT images (4-mm-thick axial slices) were obtained without intravenous contrast administration, with the patient supine, using a cerebral parenchymal window with a width of 80 Hounsfield units (HU) and center of 35 HU. 

Two experienced neuroradiologists analyzed the CT and MR images independently, and discrepancies were resolved by consensus. The following radiological features were analyzed: craniofacial disproportion with redundant scalp, occipital prominence, intracranial calcifications, simplified gyral pattern, presence of complete opercular opening, corpus callosum dysgenesis, ventriculomegaly, cerebellar hypoplasia, and pontine hypoplasia. Intraventricular septations and periventricular cysts were also noted. The criteria used to define and characterize these features were the same as those used in similar studies of congenital ZIKV syndrome^2^ ^-^ ^8^.

Craniofacial disproportion with redundant scalp was defined as a reduction of the cephalic perimeter with preservation of the facial dimensions associated with skin folds in the occipital and nuchal regions. Occipital prominence was defined as the presence of occiput protuberance. Calcifications in the brain parenchyma were recorded as present in cases with punctiform or linear calcific foci on CT, or hyperintense foci on T1 sequences and/or foci of signal drop on T2 gradient-echo MR sequences. These calcifications were located on the cortical-subcortical junction, basal ganglia, thalamus, periventricular region, and/or brainstem/cerebellum. The gyral pattern was classified as moderately simplified (pachygyria) or extensively simplified (lissencephaly with or without pachygyria). Full opercular opening was considered when at least one insula was completely exposed to the cerebrospinal fluid space, without temporal lobe coverage. Dysgenesis of the corpus callosum was recorded as present when hypoplasia or agenesis was observed. Ventriculomegaly was characterized as diffuse, when it compromised the entire extension of the lateral ventricles, or colpocephalic, when it involved only the posterior portions of the lateral ventricles, ectasia. Cerebellar hypoplasia was recorded as hemispherical, vermian, or diffuse. It was considered to be hemispherical when the cerebellar hemisphere crossed only one of the following two imaginary lines drawn in the sagittal plane: a superior line at the level of the superior colliculus of the midbrain, and an inferior line at the level of the obex. Vermis cerebellar hypoplasia was recorded when a fastigial angle > 90° was observed. Diffuse cerebellar hypoplasia was defined as the co-occurrence of vermian and hemispherical hypoplasia. Pontine hypoplasia was recorded as present when reduction of the longitudinal pons/midbrain ratio (to <1.7) and the plane of the anterior convexity of the pons was observed. 

## RESULTS

### Patient characteristics

Images from 34 patients with congenital ZIKV syndrome were evaluated; 18 (52.9%) patients were female and 16 (47.1%) were male. Patient age ranged from 1 to 14 months (mean, 4.2 months; median, 3 months). Gestational age at birth ranged from 27 to 36 gestational weeks. Birth weight ranged from 0.9 kg to 2.6 kg. Only 2 of the 34 (6%) deliveries occurred vaginally, and the remainder were interrupted by cesarean section (94%). No diagnostic imaging method was performed immediately after birth.

### Radiological findings

**FIGURE 1:**
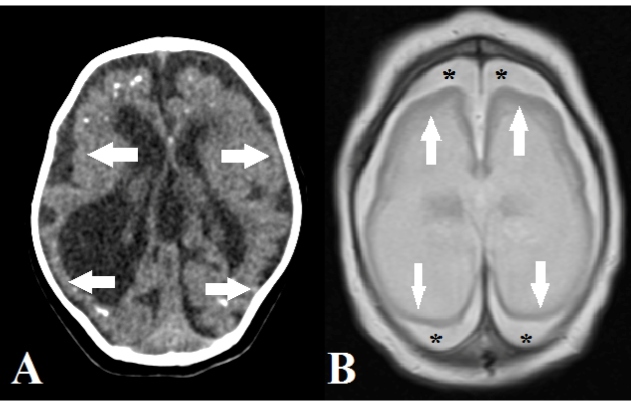
Axial computed tomography **(A)** and T2-weighted magnetic resonance **(B)** images showing marked simplification of the gyral pattern, with agyria (arrows). Note the diffuse increase in the extra-axial cerebrospinal fluid space (B; asterisks).

**FIGURE 2:**
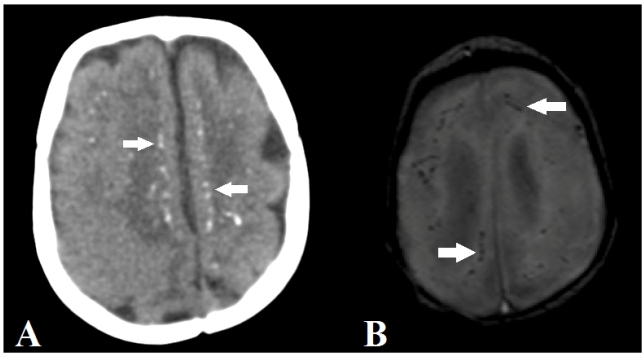
Axial computed tomography (A) and magnetic susceptibility sequence (B) images showing calcifications restricted to the cortical-subcortical junction (white arrows).

**FIGURE 3:**
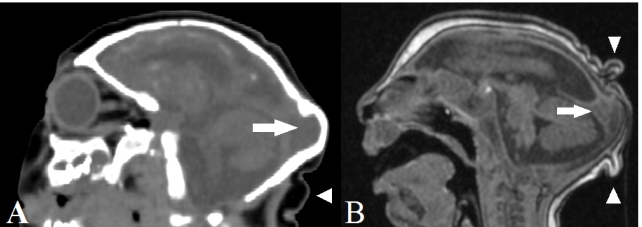
Sagittal computed tomography **(A)** and T1-weighted magnetic resonance **(B)** images showing craniofacial disproportion with a microcephalic aspect, occipital prominence (arrow), and cutis verticis gyrata (arrowheads).

**FIGURE 4:**
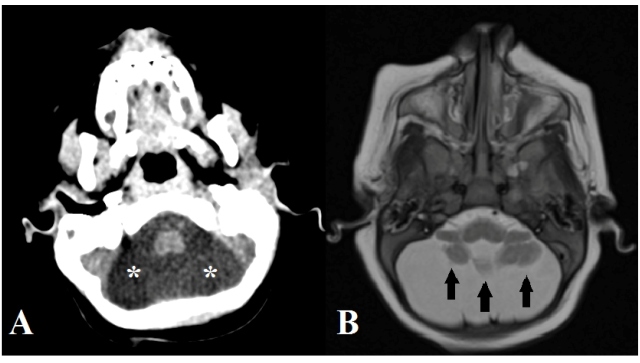
Axial computed tomography **(A)** and T2-weighted magnetic resonance **(B)** images demonstrating diffuse cerebellar hypoplasia (asterisks in A and black arrows in B).

The most common radiological findings were a simplified gyral pattern (*n* = 33, 97.0%; Figure 1) and calcifications (*n* = 32, 94.1%; Figure 2), followed by ventriculomegaly (*n* = 29, 85.3%), dysgenesis of the corpus callosum (*n* = 29, 85.3%), craniofacial disproportion and redundant scalp (*n* = 27, 79.4%), complete opercular opening (*n* = 27, 79.4%), occipital prominence (*n* = 15, 44.1%; Figure 3), cerebellar hypoplasia (*n* = 5, 14.7%; Figure 4), and pontine hypoplasia (*n* = 4, 11.8%). The results are shown in Table 1. The gyral pattern was moderately simplified in 5 (14.7%) patients and extensively simplified in 28 (82.4%) patients. Calcifications were located at the cortical-subcortical junction in 30 (88.2%) patients, in the basal ganglia in 18 (52.9%) patients, thalamus in 12 (35.3%) patients, brainstem/cerebellum in 5 (14.7%) patients, and in the periventricular region in 3 (8.8%) patients. Calcifications at the cortical-subcortical junction were found in the parietal lobe in 30 (88.2%) patients, frontal lobe in 29 (85.3%) patients, temporal lobe in 26 (76.5%) patients, and occipital lobe in 20 (58.8%) patients. Ventriculomegaly was colpocephalic in 17 (50%) patients and diffuse in 12 (35.3%) patients. Cerebellar hypoplasia affected the vermis in one (2.9%) patient, the cerebellar hemispheres in three (8.8%) patients, and was diffuse in one (2.9%) patient. Intraventricular septations and periventricular cysts were each found in one (2.9%) patient.

**TABLE 1:** Compar**i**son of CT and MRI radiological findings.

Findings	CT	MRI	p-value	OR	CI (95%)
Simplified gyral pattern	32	33	1	2.04	0.10 - 125.21
Parenchymal calcifications	32	30	0.67	0.47	0.04 - 3.59
Ventriculomegaly	29	29	1	1	0.20 - 4.86
Dysgenesis of the corpus callosum	27	29	0.75	1.49	0.36 - 6.74
Craniofacial disproportion and redundant scalp	27	27	1	1	0.26 - 3.85
Full opercular opening	27	27	1	1	0.26 - 3.85
Occipital prominence	15	15	1	1	0.34 - 2.90
Cerebellar hypoplasia	5	5	1	1	0.21 - 4.86
Pontine hypoplasia	4	4	1	1	0.17 - 5.90

N = 34 patients. **MRI:** Magnetic Resonance Imaging; **CT:** Computed Tomography; **OR:** Odds Ratio; **CI:** Confidence Interval.

One patient with marked loss of cerebral parenchyma had diffuse compensatory hydrocephalus, with the maintenance of a normal cephalic perimeter. Another patient with a normal cephalic perimeter had typical characteristics of congenital ZIKV syndrome, including a simplified but less exuberant gyral pattern and calcifications at the cortical-subcortical junction.

Despite inherent differences in the methods, both CT and MR imaging were able to identify the parameters analyzed. However, CT was able to better identify calcifications (94.1% × 88.2%), while MR imaging presented better spatial resolution for the characterization of the gyral pattern (97% x 94.1%) and corpus callosum dysgenesis (85.3% × 79.4%), but this difference was not statistically significant. Regarding the other parameters analyzed (ventriculomegaly, craniofacial disproportion and redundant scalp, complete opercular opening, occipital prominence, and cerebellar and pontine hypoplasia), there was no variation in the frequency of the findings when comparing CT and MR imaging. For statistical analysis, Fisher's exact test was performed to compare the two groups (CT and MR), using a 95% level of significance (Table 1).

## DISCUSSION

Although imaging findings are not pathognomonic, the combined consideration of neuroimaging features and clinical history may suggest congenital impairment by ZIKV. The most commonly reported findings were microcephaly, a simplified gyral pattern, calcifications, ventriculomegaly, and dysgenesis of the corpus callosum. Other observed alterations are occipital prominence, cerebellar hypoplasia, and pontine hypoplasia^2^ ^-^ ^20^. The simplified gyral pattern is likely the result of the disruption of neuronal proliferation and induction of neuronal progenitor cell apoptosis^2^ ^-^ ^20^. This was the predominant finding in our sample, present in 97.0% of cases and was extensively simplified in the majority (82.3%) of cases, reflecting the presence of lissencephaly or lissencephaly with focal areas of pachygyria. MR imaging was able to better identify the simplification of the gyral pattern in only one case, but there was no statistical significance when comparing CT and MR imaging.

Parenchymal calcifications are common in patients with congenital ZIKV syndrome and they exhibit a predilection for the cortical-subcortical junction, suggesting vascular involvement by the infection^1^ ^-^ ^16^ ^,^ ^19^ ^-^ ^20^. We identified calcifications in 94.1% of patients and localization at the cortical-subcortical junction in 88.2% of cases; calcification was thus more common in the parietal and frontal lobes, as in other studies^2^ ^,^ ^3^ ^,^ ^5^ ^,^ ^7^ ^,^ ^16^. Other sites, in decreasing order of involvement, were the nucleocapsular region, thalamus, brainstem/cerebellum, and periventricular region. With the exception of periventricular involvement, all findings were in accordance with the literature. The discordant finding of periventricular calcification may be due to hydrocephalus causing the lateral ventricles to approach the cortical-subcortical junction, which, in association with the simplified gyral pattern, may lead to confusion and misunderstanding of the correct topography. CT, as expected, was superior in the identification of calcifications when compared to the magnetic susceptibility sequences of MR imaging. This allowed for the identification of two cases that were negative for parenchymal calcifications on MR imaging. However, when comparing the methods, there was no statistically significant difference. Our study focused only on detecting whether parenchymal calcifications were present or not. A limitation of our study is that the sensitivity of CT and MR imaging in detecting parenchymal calcifications in each affected region (cortical-subcortical junction, nucleus-capsular region, thalamus, and periventricular and infratentorial regions) was not compared.

Ventriculomegaly is a frequently reported finding in congenital ZIKV syndrome^2^ ^,^ ^4^ ^-^ ^8^ ^,^ ^15^ ^,^ ^16^ ^,^ ^19^ ^,^ ^20^ and is commonly related to the destruction of the cerebral parenchyma. We observed ventriculomegaly in 85.3% of patients, with a predominance of the colpocephalic pattern (50% of cases). The presence of intraventricular septations and periventricular cysts has been described in the literature^17^, but as infrequent; in agreement with this characterization, we observed each of these conditions in only one patient. Regarding the ability to detect ventriculomegaly, CT and MR imaging showed similar results.

In dysgenesis of the corpus callosum, agenesis may be related to the direct deleterious effect of ZIKV infection and the production of pro-inflammatory cytokines, and hypoplasia may be related to the damage done to the cortical development and consequent reduction of interhemispheric fibers^2^ ^-^ ^9^ ^,^ ^12^ ^,^ ^14^ ^,^ ^16^ ^,^ ^20^ ^-^ ^26^. We found dysgenesis of the corpus callosum in 85.3% of patients, similarly to previous reports^2^ ^,^ ^3^ ^,^ ^5^ ^,^ ^7^. MR imaging was able to better identify the dysgenesis of the corpus callosum in two cases, but there was no statistically significant difference between the two methods.

We observed craniofacial disproportion and redundant scalp in 79.4% of patients, in agreement with other studies. Craniofacial disproportion develops with the destruction of the cerebral parenchyma, with consequent microcephaly and the preservation of facial growth. In all cases in this study, it was associated with redundant scalp. Redundant scalp, often reported in patients with congenital ZIKV syndrome, is rare with other causes of microcephaly and results from interruption of the growth of the brain while the development the skull and skin is maintained^2^ ^,^ ^3^ ^,^ ^5^ ^,^ ^7^. Our findings corroborated previous observations of normal head circumference in patients with congenital ZIKV syndrome^2^ ^-^ ^8^. CT and MR imaging showed similar ability to detect craniofacial disproportion and redundant scalp.

We observed full opercular opening (complete insula exposure) in 79.4% of patients, in direct correlation with the presence of an extensively simplified gyral pattern. We did not find data on complete opercular opening in the literature, but this feature may serve as a marker of this gyral pattern. CT and MRI showed no difference in the ability to detect the complete opercular opening.

Occipital prominence, found in 44.1% of patients, has been described in cases of ZIKV-related microcephaly^2^ ^,^ ^5^ ^-^ ^8^ ^,^ ^19^. It is characterized by the maintenance of occipital bone growth with the collapse of the rest of the skull cap due to the destruction of the cerebral parenchyma. We did not find data on occipital prominence in this context in the literature. In addition, as demonstrated, both CT and MRI can detect this finding, with no statistically significant difference.

Cerebellar and pontine hypoplasia are infrequent in patients with congenital ZIKV infection; they demonstrate the preferential involvement of the supratentorial compartment by ZIKV. We observed cerebellar hypoplasia in 14.7% of patients and pontine hypoplasia in 11.8% of patients; this greater involvement of the cerebellum than the pons is in agreement with most previous reports, but the frequencies were lower in our sample than reported previously^2^ ^,^ ^3^ ^,^ ^5^ ^,^ ^7^. This difference may be due to our use of more than one parameter (i.e., more rigorous criteria) to characterize hypoplasia. Regarding the ability to detect cerebellar and pontine hypoplasia by the methods analyzed, there was no difference between CT and MR imaging.

As expected, due to the superior capacity of CT to detect calcium deposits, parenchymal calcifications were better individualized than with MR. However, due to the better spatial resolution of MR, it was able to evaluate the gyral pattern and callosal involvement more precisely. However, in the evaluation of calcifications as well as the gyral pattern and callosal involvement, there was no statistical difference in the detection capacity of CT and MR. These data demonstrate that CT is as capable as MR in assessing brain malformations in children with congenital ZIKV syndrome, which is important information since it is an easier method to access and requires a much shorter examination time. In addition, sedation is not necessary in CT, while it is mandatory in MR.

This study had some limitations. Our sample comprised only 34 patients, and diagnoses were based on suggestive clinical histories in association with compatible radiological and serological findings. Reverse-transcription polymerase chain reaction analysis was not performed in any case. In addition, due to the complexity of our center, most cases may have been referred because of suspected serious involvement, with underrepresentation of milder cases. Our patients may have had other unconfirmed or as-yet unidentified infections and syndromes. However, few other studies of congenital ZIKV syndrome have included similar numbers of cases, and we found no study in which the distribution of calcifications was evaluated by lobe, or in which complete opercular opening and occipital prominence were evaluated.

The main findings in congenital ZIKV syndrome are craniofacial disproportion with a microcephalic aspect, accompanied by calcifications (predominantly at the cortical-subcortical junction), malformations of cortical development, ventriculomegaly, and abnormalities in the formation of the corpus callosum. However, attention should be paid to the spectrum of potential presentations of congenital ZIKV syndrome, and the possibility of ZIKV involvement should not be ruled out when microcephaly is not present or when the neuroimaging findings are more subtle.

In addition, we demonstrated that there was no statistical difference in the detection of congenital ZIKV syndrome-associated brain malformations between CT and MR. Therefore, CT might be preferrable for the diagnosis of these patients as it is an exam that is easier to access, faster, less costly and does not require sedation.
